# The C-Reactive Protein–Triglyceride–Glucose Index as a Biomarker of Metabolic Dysfunction-Associated Steatotic Liver Disease: Results from the Population-Based Italian NUTRI-HEP Cohort

**DOI:** 10.3390/biomedicines14081860

**Published:** 2026-08-19

**Authors:** Gianluigi Casimo, Rossella Donghia, Caterina Bonfiglio, Rossella Tatoli, Giovanni Maria Biancofiore, Gianluigi Giannelli

**Affiliations:** 1 Data Science Unit, National Institute of Gastroenterology-IRCCS “Saverio de Bellis”, Castellana Grotte, 70013 Bari, Italy; rossella.donghia@irccsdebellis.it (R.D.); catia.bonfiglio@irccsdebellis.it (C.B.); rossella.tatoli@irccsdebellis.it (R.T.); giovanni.biancofiore@irccsdebellis.it (G.M.B.); 2 Scientific Direction, National Institute of Gastroenterology-IRCCS “Saverio de Bellis”, Castellana Grotte, 70013 Bari, Italy; gianluigi.giannelli@irccsdebellis.it

**Keywords:** MASLD, CTI index, C-reactive protein, biomarker, liver, steatosis

## Abstract

**Background/Objectives**: Metabolic Dysfunction-Associated Steatotic Liver Disease (MASLD) is rapidly becoming one of the main Non-communicable Chronic Diseases (NCDs) in the global population. It is based on the presence of hepatic steatosis linked to other metabolic alterations. Currently, the gold standard techniques to evaluate hepatic steatosis are liver biopsy and hepatic ultrasound examinations (FIBROSCAN^®^). However, liver biopsy is an invasive technique and ultrasonic examination is expensive. Therefore, the search for a possible low-cost and non-invasive examination has highlighted the C-reactive protein-triglycerides-glucose index (CTI index) as a possible alternative. This index has already been shown to be associated with cancer survival and with cardiovascular diseases and stroke in previous studies and was found to be predictive for the development of several cardiovascular diseases and stroke incidence. This analysis aimed to investigate the possible association between CTI and MASLD in a southeastern Italian cohort. **Methods**: This study included 1297 subjects from the NUTRI-HEP project: 629 (48.5%) were affected by MASLD and 668 (51.5%) were not. The CTI index was calculated using the formula proposed by Ruan et al. and was used as both a continuous and a categorical variable. Four logistic regression models were fitted using MASLD as the outcome and the CTI index as the exposure. They were adjusted for several covariates. **Results**: All logistic regression models indicated a positive association between the CTI index and MASLD development. Focusing on Model 4, using Q1 as a reference, the Odds Ratios were 1.48 (95% C.I. 1.00; 2.17, *p* = 0.049), 2.06 (95% C.I. 1.38; 3.08, *p* < 0.001) and 3.62 (95% C.I. 2.32; 5.64, *p* < 0.001). **Conclusions**: This study highlights a strong association between MASLD and the CTI index. This association strengthens the role of CTI in predicting the onset and development of MASLD.

## 1. Introduction

Non-communicable Chronic Diseases (NCDs) are rapidly becoming the major class of diseases affecting people across the globe. A large proportion of these NCDs target the liver as the main organ, known as steatotic liver diseases (SLDs) [1].

In particular, Metabolic Dysfunction-Associated Steatotic Liver Disease (MASLD), the most common SLD, is defined as fat accumulation in hepatocytes, linked to one or more of the following metabolic dysfunctions: body mass index higher than 25 kg/m^2^, a concentration of glucose over 100 mg/dL in the bloodstream, blood pressure over 130/85 mmHg, and dyslipidemia produced by a concentration of triglycerides over 150 mg/dL or HDL levels under 40 mg/dL in men and 50 mg/dL in women [2]. Moreover, it is mandatory that people defined as MASLD-positive patients must have a limited intake of alcohol during the day. This definition was proposed in the Delphi process in 2023 [1], to expand the implication of steatosis not only to the liver but to the whole organism [1].

Approximately 38% of the adult population is currently affected by MASLD [3,4,5] (2016–2019) with a 50% increase in the past 20 years [3], when the prevalence was estimated to be around 25% in adults [4].

Currently, the main exams used to estimate liver fat accumulation are liver biopsy and ultrasonic elastography [6]. The first is considered the gold standard for estimating hepatic lipid accumulation, despite being an invasive technique and unsuitable for large-scale screening [7]. The latter is far less invasive than biopsy but is considered more expensive and, because of that, less accessible.

Due to these downsides of liver biopsy and ultrasonic elastography, researchers have started to investigate common biomarkers as possible indicators associated with MASLD status. The C-reactive protein–triglyceride–glucose (CTI) index has gained popularity in this field of research because of its design [8].

The CTI index was originally proposed by Ruan et al. [7] as a measurement to predict the probability of survival in patients with cancer. It was designed starting from a preexisting index, the triglycerides-glucose index (TyG), which has been considered a good estimator of Insulin Resistance in many cancer-related studies [8]. In addition to TyG, the CTI index includes the concentration of C-Reactive Protein. Ruan et al. integrated it into their design because of its association with tumor progression and survival prediction [8,9].

C-reactive protein is considered one of the main markers of inflammation in the bloodstream because of its increased concentration during these stressful states [10]. Therefore, owing to the ubiquity of CRP in inflammation pathways and the central role of IR in many NCDs, many studies have investigated the association of the CTI index with several non-communicable diseases, such as cardiovascular diseases and stroke [11,12,13]. Some studies have investigated the association between the CTI index and the presence of MASLD [7,14,15,16,17] due to the central role of IR in the definition of this disease and the inflammatory status generated by fat accumulation in the liver.

Based on this understanding, we decided to estimate the CTI index value within the NUTRIHEP cohort located in Putignano (Bari), in southeastern Italy. We then investigated the potential relationship between MASLD and CTI measurements. Up to now, there are no studies in the scientific literature that examine this association in a comparable population.

## 2. Materials and Methods

### 2.1. Study Population

The research proposed in this article is based on the NUTRI-HEP cohort study of the Putignano citizenry, which was initiated in 2005–2006. General Practitioners’ (GP) registers were used instead of census records to enlist the participants, as there were no notable differences in the age-gender distribution between the two records. In particular, individuals over the age of 18 years were systematically selected at random [18]. The participants attempted a first follow-up recall between 2015 and 2018; a total of 1426 responded. The analysis explained in this study is based on follow-up data. All participants signed an informed consent form and received detailed information about the medical data to be studied. The cohort study was approved by the Ethical Committee of the Minister of Health (DDG-CE-792/2014, on 14 February 2014).

### 2.2. Data Collection

Expert physicians and/or nutritionists interviewed the participants regarding their sociodemographic framework, lifestyle habits, health status, and clinical history. The Q&A session included questions on current or past use of tobacco, alcohol intake, food consumption, educational level [19], work [20], and marital status. The subjects’ weight and height were measured while wearing only underclothing and no shoes, using an electronic balance (SECA©, Hamburg, Germany) to record weight to the nearest 1 kg, and a wall-mounted stadiometer (SECA©, Hamburg, Germany) to measure height to the nearest 1 cm. Systolic and Diastolic pressures (SBP and DBP) were measured following international guidelines [21,22] and, to reduce operators’ reading errors, an average of three measurements was calculated. Moreover, the following blood parameters were collected using the COBAS 8000 autoanalyzer (ROCHE Diagnostics SPA, Monza, Italy): fasting serum glucose (FSG), fasting insulin (FI), Hemoglobin A1c (HbA1c), triglycerides, total cholesterol, low-density lipoprotein cholesterol (LDL-C), High-density lipoprotein cholesterol (HDL-C), Aspartate Transferase (AST), Alanine Transferase (ALT), Gamma-Glutamyl Transferase (GGT), ferritin, and C-reactive protein.

Insulin resistance was estimated using the Homeostasis Model Assessment of Insulin Resistance (HOMA-IR) formula [23]:

(1) HOMA−IR= FSG(mgdL)×FI(μUmL)405.

Participants underwent standardized ultrasound examinations using a Hitachi H21 Vision with a 3.5 MHz transducer (Hitachi Medical Corporation, Tokyo, Japan) to examine the liver parenchyma. The hepatic fat content was evaluated using a scoring system that provided a semi-quantitative assessment. The extent of hepatic fat infiltration was evaluated based on liver echotexture, hepatic echo penetration, clarity of hepatic blood vessels, and differentiation of the hepatic diaphragm in the echo amplitude [24]. Table S1 shows the ultrasound board used in this study to determine the steatosis grade (see Supplementary Materials).

The subjects self-completed the European Prospective Investigation into Cancer and Nutrition (EPIC) Food Frequency Questionnaire (FFQ) [25,26,27] with their eating habits and returned it after one week. Nutritionists validated all responses and uploaded the questionnaire into a custom online tool to convert the entered nutritional data into micro- and macronutrient categories and grams for each food type.

### 2.3. Outcome Assessment

The MASLD definition provides for hepatic steatosis combined with at least one of the following cardiometabolic risk factors:

1. Body Mass Index (BMI) > 25 kg/m^2^ or waist circumference > 94 cm in men and >80 cm in women;
2. FSG ≥ 100 mg/dL, 2 h post-load glucose ≥ 140 mg/dL, HbA1c of ≥5.7%, or being on specific medication;
3. Blood pressure of 130/85 mmHg or higher or on specific drug treatment;
4. Serum triglyceride levels ≥ 150 mg/dL or on specific medications;
5. Serum HDL cholesterol < 40 mg/dL in men and <50 mg/dL in women, or on specific drug treatment.

Furthermore, according to the current liver disease nomenclature, MASLD was defined in subjects with an average alcohol intake ≤20 g/day in women and ≤30 g/day in men. Subjects with metabolic dysfunction and higher alcohol consumption, corresponding to 20–50 g/day in women and 30–60 g/day in men, fall within the MetALD category [28]. Other forms of liver diseases coexisting with this metabolic syndrome, such as MASLD + HCV, were ruled out to avoid altering the natural history of this metabolic disorder [29] and to reduce potential collinear problems.

### 2.4. Exposure Variables

The C-reactive protein–triglyceride–glucose index was calculated using the formula proposed by Ruan et al. [7]:

(2) $$CTI\;Index=0.412\;\times ln\;(CRP\;\left(\mathrm{mg}/L\right))+\;ln\;(\frac{TG\;\left(\mathrm{mg}/\mathrm{dL}\right)\;\times \;FPG\;(\mathrm{mg}/\mathrm{dL})}{2})$$

Therefore, where needed, the data were corrected for the right unit of measurement before calculating the final index. The CTI index was then converted from a continuous variable to a categorical one, based on quartiles, to better describe the trend analyzed in the article.

### 2.5. Confounding Variables

The confounding variables used to adjust the logistic regression models were selected based on similar studies in the literature and their correlation with MASLD and the biomarkers used in the CTI index formula. In the sociodemographic framework, we selected the following variables: gender, age (as a continuous variable), education title, work and marital status, personal annual income, and house location. Daily kilocalorie intake, alcohol consumption and smoking habits (as an answer to the question “Do you smoke on the date of the interview?”) were selected as variables describing lifestyle habits. Furthermore, HOMA-IR (as a categorical variable divided into over and under 2.5, selected as a cut-off of insulin resistance), cancer (as a categorical answer to the question “Do you suffer (or have suffered) from a diagnosed type of cancer at the date of the interview?”), hypertension (as a categorical answer to the question “Do you suffer of diagnosticated hypertension at the date of the interview?”), Total Cholesterol, Aspartate Aminotransferase (AST) and Alkaline Phosphatase (ALP) were chosen as variables to identify the clinical framework of the participants. To avoid collinearity, other significant variables were omitted from the model, such as BMI, which was left out as it was a primary metabolic alteration observed in the cohort correlated to the MASLD definition, as shown in Table S2 (see Supplementary Materials).

### 2.6. Statistical Analysis

The participants’ parameters are reported in Table 1 as frequencies and percentages (%) for categorical variables and as means and standard deviations (M ± SD) or medians and interquartile ranges for continuous variables. For continuous variables, the Wilcoxon signed-rank test and the Kruskal–Wallis test were used to compare two groups or more than two groups; for categorical variables, we used the χ^2^ or Fisher test as needed. We fitted four logistic regression models to estimate odds ratios (ORs) and 95% Confidence Intervals (CIs) with the CTI Index, both as a continuous and categorical variable, as the predictor and MASLD as the outcome.

To better estimate the odds of an event occurring in one group compared with another, the results are shown in Odds Ratios (ORs). An OR of 1 indicates no difference between the control and exposure groups, an OR lower than 1 indicates a decreased risk in the exposure group, and an OR greater than 1 suggests an increased risk [30].

The models were adjusted in the following manner: the first model was unadjusted; Model 2 was adjusted for gender and age; the third model was further adjusted also for education, work, marital status, personal annual income and house location; furthermore, the last model was also adjusted for alcohol consumption, smoking habits, daily kilocalorie intake, HOMA-IR (as a categorical variable under/over 2.5 cut-off), cancer and hypertension diagnosis, total cholesterol, Alkaline Phosphatase (ALP) and Aspartate Aminotransferase (AST). In the models using the CTI categorical variable, the estimated coefficients were transformed into odds ratios (OR), using the first quantile (0–7.88) as reference.

Confounding variables were selected from the existing literature and using the minimum absolute reduction and selection (LASSO) [31] to reduce the number of predictors and to identify the most useful ones. Moreover, the variance inflation factor (VIF) was evaluated to check multicollinearity and covariates with VIF > 5 were discarded [32] (Table S3, see Supplementary Materials).

The two-tailed probability level was set at 0.05 to test the null hypothesis of non-association. The analyses were conducted using Stata Corp. 2025. Stata Statistical Software: Release 19. StataCorp LLC. (College Station, TX, USA).

## 3. Results

The distribution of several sociodemographic and clinical factors between the CTI index quartiles, among the NUTRIHEP participants, is shown in Table 1.

The higher the CTI index, the higher the age of the subjects, suggesting a higher inflammatory status in older people. This is also confirmed by the distribution of work status among the quartiles, where pensioners are the most frequent class in Q4 (35.4%) (*p* < 0.001).

Another noteworthy stratification in the CTI index quantiles is gender; male participants tended to have a higher CTI value than females, with 51.8% in Q4 for the former against 47.2% for female participants (*p* < 0.001).

Education seems to play a protective role, as shown by Donghia et al. [32]; a higher education degree is linked to a lower CTI, while participants with a primary school degree tend to have a higher inflammatory status (*p* < 0.001).

A visible trend is observable between the marital status classes, in which the “Single” and “Separated or Divorced” classes show lower values of CTI, while the “Married or Living together” class increases in percentage across the quartiles (*p* < 0.001).

Moreover, a slight association can be made between the CTI index and the house location of the subjects (*p* = 0.477), showing that people living in the periphery of Putignano have a trend leaning toward the highest quantile, while participants living Downtown or in the countryside show a decrease in percentage from the least stressed quartile to the most stressed quartile, confirming that the neighborhood can influence the stress level of the participants [33,34], affecting their inflammatory status.

An obvious association can be made between the distribution of people with a diagnosed case of cancer (*p* = 0.004) or hypertension (*p* < 0.001); in both cases, there is a notable increasing trend in CTI values for people with an affirmative diagnosis, while in the case of a negative diagnosis, the lower quartiles are the most populated. This occurs because of the implication of C-reactive protein in most biological inflammatory pathways [10,35].

Furthermore, dietary habits are also strictly linked to CTI distribution. In fact, the fourth quartile, which is composed mainly of MASLD subjects (74.5%), has a higher intake of kilocalories during the day (*p* = 0.515) and consumes more alcohol (*p* < 0.001) than the others, resulting in a higher BMI (*p* < 0.001) over the type I obesity threshold, an increased level of total cholesterol in blood (*p* < 0.001), and a considerably higher value of HOMA-IR (*p* < 0.001) than the other quantiles over the 2.5 cut-off value used to identify a possible case of Insulin Resistance.

An additional noteworthy trend was observed in the concentration of AST (*p* < 0.001) and ALP (*p* < 0.001) in the bloodstream across the four quantiles, confirming the visible inflammation status of the liver in participants with a higher CTI index value. The same trend was observed for the biomarkers used in the CTI index formula (TG (*p* < 0.001), FSB (*p* < 0.001), and CRP (*p* < 0.001)).

No substantial stratification was found for personal annual income (*p* = 0.052) and rMED score (*p* = 0.220) [36] among the CTI index, although they notably influence the development of MASLD [37].

The distribution of these same sociodemographic and clinical factors among people affected or not by MASLD is reported in the Supplementary Materials (Table S4).

**Figure 1:**
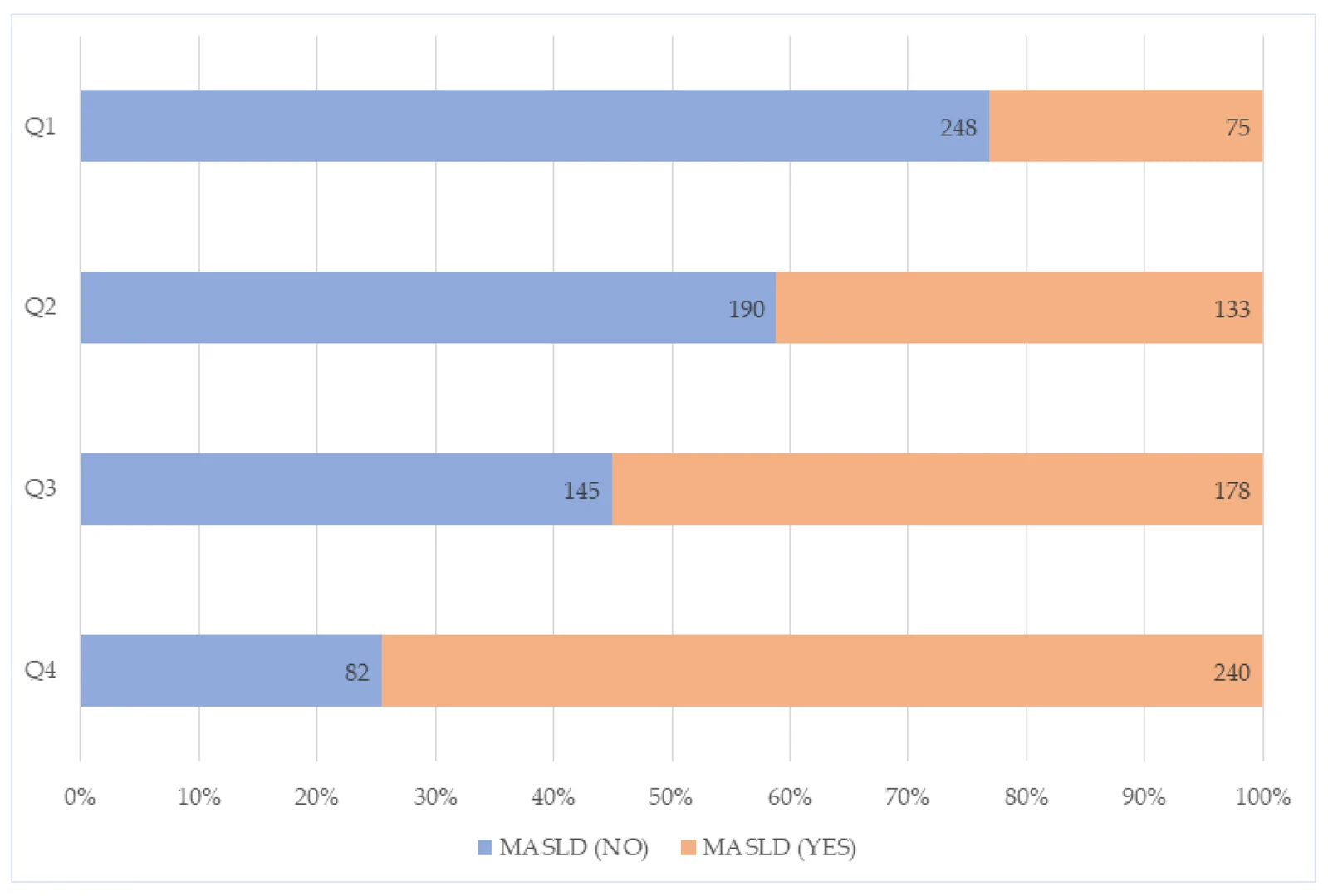
Histogram of MASLD-affected and MASLD-non-affected subjects in each CTI index quantile.

The relationship between MASLD and the CTI index is already notable in the distribution of MASLD and no MASLD subjects, as highlighted in Figure 1. In fact, there is a dominance of non-affected participants in the two lowest quartiles, while in the second two the MASLD participants exceed 50%.

**Figure 2:**
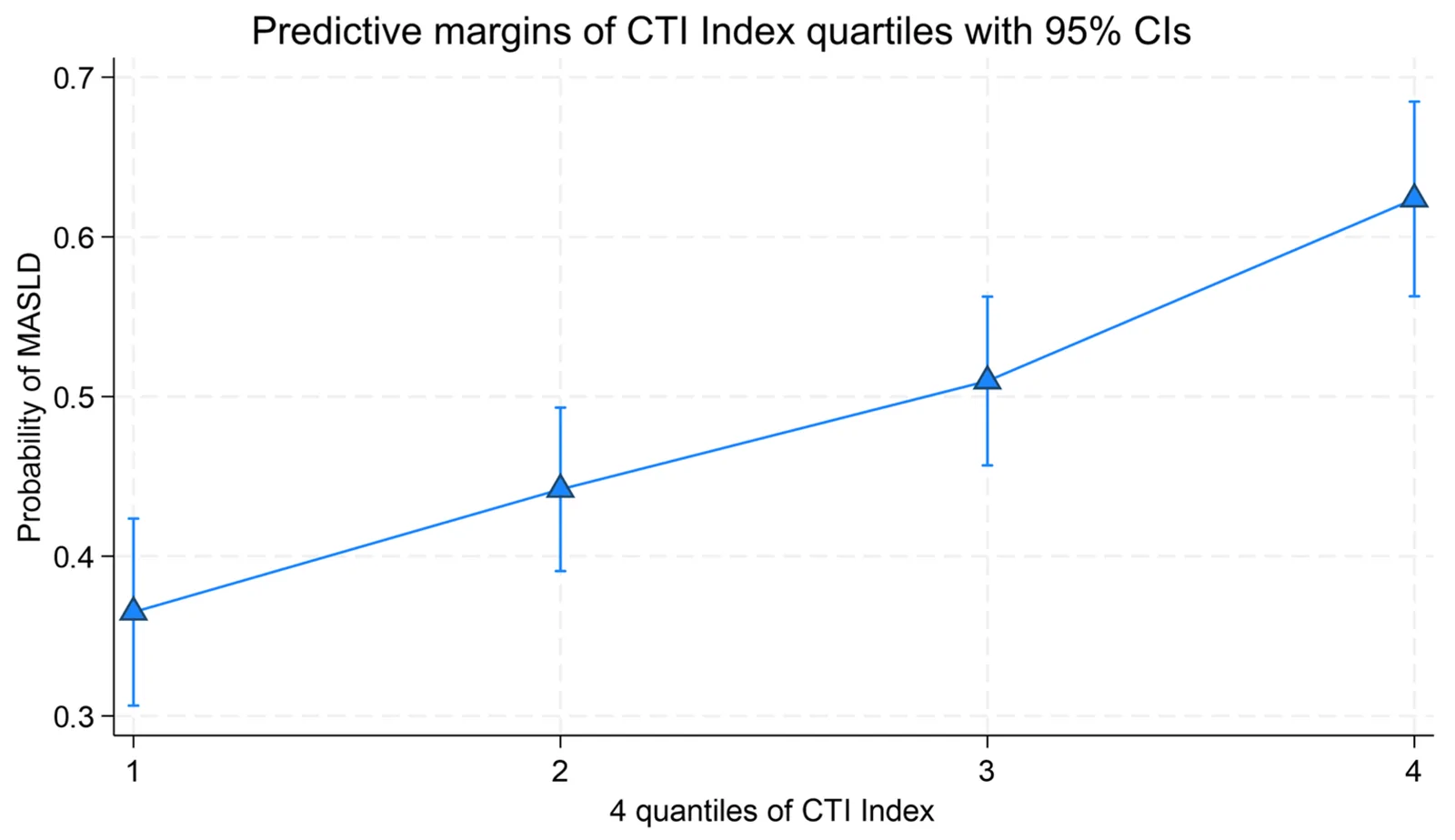
Predictive margins from the MASLD model using CTI index quantiles in Model 4.

The association between MASLD and the CTI index was estimated using four models, as shown in Table 2, considering the CTI index as a continuous or categorical variable. Each model was adjusted for several covariates, as described in the Statistical Analyses Section. The margins plot of Model 4 is presented in Figure 2. Moreover, two other models similar to Model 2 were performed (presented in Table S5, Supplementary Materials) to study the stratification of the participants by both sex and age (as categorical under or over 65 years), and the margins plots are presented in Figure S1.

A notable trend is visible among all the logistic models presented in this study, with an increase in OR—using the first quartile as a reference–from a lower value of the CTI index to a higher one.

In the unadjusted model, the association between MASLD development and the CTI index value doubled at each quartile. Starting from the second quartile (7.89–8.39) where the probability increases 2.32 times than the reference (1.65–3.25 95% C.I., *p* < 0.001), then it doubled in the next quantiles (OR = 4.06, 2.89–5.70 95% C.I., *p* < 0.001), arriving to a probability more than nine times higher than the reference quartile in the Q4 (OR = 9.68, 6.75–13.87 95% C.I., *p* < 0.001).

After adjusting for gender and age in the logistic regression, a drop in the O.R. value was observed owing to the higher probability of older adults and males developing chronic liver diseases. In fact, Q2 drops to a probability 61% higher than the first quartile (OR = 1.61 1.12–2.31 95% C.I., *p* < 0.05); the third quartile shows an Odd Ratio of 2.50 compared with the reference (1.74–3.60 95% C.I., *p* < 0.001); and, finally, the last quartile has more than 5 times the probability of developing MASLD than the lowest one (OR = 5.61 3.83–8.23 95% C.I., *p* < 0.001). This significant drop in the odds ratio, linked to age and gender of the participants, is confirmed by the margins plots in Figure S1, stratified by gender and age (over/under 65 years old) as described above.

In Model 3, an additional correction made for sociodemographic parameters showed a slight adjustment for the Odds Ratio, with a decrease for each quartile: 1.47, 2.38, and 5.16, respectively. This is due to the influence of educational level, type of work, income, and house location [33,34,37,38].

Finally, in the last model, the correction for clinical history and lifestyle habits highlighted another decrease in the OR values, with a probability 48% higher in the second quartile (OR = 1.48 1.00–2.17 95% C.I., *p* < 0.05) than the reference; an OR two times higher in Q3 (OR = 2.06 1.38–3.08 95% C.I., *p* < 0.001) than Q1 and a probability of developing MASLD 3.62 times than Q1 in the last quartile (OR = 3.62 2.32–5.64 95% C.I., *p* < 0.001).

## 4. Discussion

The models presented above show that in the NUTRIHEP cohort, a strong positive correlation between the probability of developing MASLD and the increase in CTI index values can be observed.

This strict association originates from the nature of the CTI index itself: it is based on the TyG (triglycerides–glucose) index, which has already been used to analyze many metabolic dysfunctions, in particular insulin resistance linked to T2DM [16], which is one of the main co-causes of MASLD, due to its facilitation of hepatic lipid accumulation [14]. Furthermore, the inclusion of C-reactive protein allows the index to cover the chronic inflammatory status caused by fat accumulation in the liver. The expression of this protein mainly occurs in hepatic cells after the activation of inflammatory signaling pathways and increased production of inflammatory cytokines. These molecules are responsible for the enhanced expression of the *CRP gene*; in particular, interleukin-6 (IL-6) is the main cytokine linked to the expression of C-reactive protein in hepatocytes [10]. C-reactive protein has also been identified as a strong independent predictor of cardiovascular diseases, which are linked to the onset of MASLD [10,39].

Therefore, owing to these characteristics, CTI measurement can describe the complex interactions between metabolic disorders and inflammation pathways, which are typical of MASLD.

An additional methodological consideration is the partial overlap between the CTI index and the diagnostic criteria for MASLD. Specifically, fasting glucose and triglyceride levels are components of the CTI index and represent two of the five cardiometabolic criteria included in the current MASLD definition. This overlap may have contributed, at least in part, to the observed associations. However, MASLD was not defined solely by these biomarkers but required the presence of ultrasound-detected hepatic steatosis together with at least one of five cardiometabolic risk factors, while CRP was not included in the diagnostic criteria. Therefore, although a degree of conceptual overlap exists, the CTI index and MASLD definition are not mathematically equivalent. Consequently, the observed association should be interpreted in light of this partial overlap.

This analysis is in line with previous studies [7,14,15,16,17,40,41,42] in which a similar correlation between the novel CTI index and MASLD was found. This literature can open the application of this and other indices, based on biomarkers, as predictors of the development of chronic diseases. The use of a biomarker-based index has advantages over both of the two main techniques of steatosis validation. It allows for a lower processing time than biopsy, which is referred to as the gold standard. Compared with hepatic ultrasound examinations, the biomarker-based index is less expensive because it is based on blood parameters that can be easily collected with a routine laboratory blood test; this could allow the analysis of MASLD in Countries where there is no access to the two techniques mentioned above [7,12,40]. Moreover, due to the absence of a proper drug treatment for MASLD, a faster and easier way to predict its status could allow better tracking of its progression, and, because of that, a quicker intervention in correcting the dietary and lifestyle habits and reducing the probability of MASLD evolving into much more severe diseases (e.g., fibrosis and HCC) [14,15].

The stratified analysis, shown in Figure S1, highlighted a clear difference in the probability of developing MASLD between individuals under and over 65 years of age within the same CTI index quartiles. This confirms a higher susceptibility of older subjects to the onset of MASLD than younger subjects, with equal CTI values. Based on these results, the CTI index may represent a useful biomarker associated with MASLD. Age-stratified analyses suggested a stronger association among participants aged ≥65 years. However, these findings should be considered exploratory, although dedicated diagnostic accuracy studies are required before considering its use as a screening tool for older and more fragile individuals, rather than other more invasive tests.

Finally, the trend remained stable despite the adjustment for several other diseases linked to inflammatory pathways, allowing us to confirm that this measurement can identify not only inflammatory status given by cancer, diabetes, or cardiovascular diseases [8,11,12,13,43], but it can also detect a stressful status of the organism given by an abnormal deposit of fat in the liver and the metabolic disorders linked to it.

### Strengths and Limitations

The study presented in this article is centered on a large cohort of Italian participants from southeastern Italy. This strengthens the analysis, as it is characterized by strong adherence to the Mediterranean Diet, which is recognized as one of the most effective dietary patterns for combating metabolic syndromes [43]. Additionally, few studies have examined this index in a similar cohort. However, at the time the screening analysis was collected, the laboratory did not have available kits to estimate the concentration of high-sensitivity C-reactive protein; therefore, the results presented in this article are based on C-reactive protein. This could result in possible variations in the evaluation of the linear regression between the CTI index and MASLD. Moreover, the cross-sectional design of this study only demonstrates a strict association between the onset of MASLD and the increase in the CTI index; therefore, further detailed studies must be conducted to identify a possible use of this measurement as an estimation of MASLD status.

## 5. Conclusions

In conclusion, the research presented in this article identifies a positive association between the probability of developing MASLD and a higher CTI index value in the NUTRIHEP cohort, particularly in individuals aged >65 years. Confirming the findings of similar studies conducted on far different cohorts, such as CHARLES and NHANES, this study suggests that CTI may represent a complementary biomarker associated with MASLD rather than an alternative to current diagnostic methods.

## Figures and Tables

**Table 1:** Demographic and clinical characteristics of subjects stratified for quartiles of CTI index (n = 1291).

Parameters *	Quantiles of CTI Index					
	1 (*n* = 323)	2 (*n* = 323)	3 (*n* = 323)	4 (*n* = 322)	Total (*n* = 1291)	*p* ^
Age	45.65 (12.44)	54.23 (13.94)	57.60 (14.04)	59.87 (12.69)	54.33 (14.34)	<0.001 ^†^
MASLD (%)						<0.001
No	248 (76.8)	190 (58.8)	145 (44.9)	82 (25.5)	665 (51.5)	
Yes	75 (23.2)	133 (41.2)	178 (55.1)	240 (74.5)	626 (48.5)	
Sex (%)						<0.001
Female	228 (70.6)	191 (59.1)	173 (53.6)	152 (47.2)	744 (57.6)	
Male	95 (29.4)	132 (40.9)	150 (46.4)	170 (52.8)	547 (42.4)	
Education (%)						<0.001
Primary school	28 (8.7)	58 (18.0)	86 (26.6)	108 (33.5)	280 (21.7)	
Secondary school	79 (24.5)	103 (31.9)	104 (32.2)	96 (29.8)	382 (29.6)	
High School	149 (46.1)	120 (37.2)	97 (30.0)	91 (28.3)	457 (35.4)	
Graduated	67 (20.7)	42 (13.0)	36 (11.1)	27 (8.4)	172 (13.3)	
Work (%)						<0.001
Managers & Professionals	28 (8.7)	24 (7.4)	25 (7.7)	25 (7.8)	102 (7.9)	
Craft, Agricultural and Sales Workers	147 (45.5)	120 (37.2)	104 (32.2)	96 (29.8)	467 (36.2)	
Elementary Occupations	55 (17.0)	46 (14.2)	42 (13.0)	40 (12.4)	183 (14.2)	
Housewife	30 (9.3)	39 (12.1)	39 (12.1)	33 (10.2)	141 (10.9)	
Pensioners	32 (9.9)	80 (24.8)	97 (30.0)	114 (35.4)	323 (25.0)	
Jobless	31 (9.6)	14 (4.3)	16 (5.0)	14 (4.3)	75 (5.8)	
Marital Status (%)						<0.001
Single	72 (22.3)	51 (15.8)	31 (9.6)	26 (8.1)	180 (13.9)	
Married or Living together	239 (74.0)	247 (76.5)	272 (84.2)	271 (84.2)	1029 (79.7)	
Separated or Divorced	9 (2.8)	8 (2.5)	6 (1.9)	5 (1.6)	28 (2.2)	
Widower	3 (0.9)	17 (5.3)	14 (4.3)	20 (6.2)	54 (4.2)	
Personal annual income (%)						0.052
<5000	2 (0.6)	0 (0.0)	1 (0.3)	0 (0.0)	3 (0.2)	
5000–10,000	5 (1.5)	13 (4.0)	11 (3.4)	14 (4.3)	43 (3.3)	
10,000–20,000	95 (29.4)	92 (28.5)	120 (37.2)	121 (37.6)	428 (33.2)	
20,000–30,000	138 (42.7)	131 (40.6)	114 (35.3)	120 (37.3)	503 (39.0)	
30,000–40,000	36 (11.1)	43 (13.3)	38 (11.8)	29 (9.0)	146 (11.3)	
40,000–50,000	18 (5.6)	19 (5.9)	10 (3.1)	9 (2.8)	56 (4.3)	
>50,000	7 (2.2)	2 (0.6)	8 (2.5)	4 (1.2)	21 (1.6)	
No answer	0 (0.0)	0 (0.0)	1 (0.3)	0 (0.0)	1 (0.1)	
Do not know	22 (6.8)	23 (7.1)	20 (6.2)	25 (7.8)	90 (7.0)	
House Location (%)						0.477
Downtown	109 (37.3)	131 (43.1)	117 (39.0)	117 (39.3)	474 (39.7)	
Periphery	131 (44.9)	132 (43.4)	145 (48.3)	141 (47.3)	549 (46.0)	
Countryside	52 (17.8)	41 (13.5)	38 (12.7)	40 (13.4)	171 (14.3)	
Smoke (%)						0.784
No	288 (89.2)	282 (87.3)	284 (87.9)	278 (86.6)	1132 (87.8)	
Yes	35 (10.8)	41 (12.7)	39 (12.1)	43 (13.4)	158 (12.2)	
Cancer (%)						0.004
No	297 (98.3)	300 (96.2)	282 (93.1)	289 (93.8)	1168 (95.3)	
Yes	5 (1.7)	12 (3.8)	21 (6.9)	19 (6.2)	57 (4.7)	
Hypertension (%)						<0.001
No	267 (88.4)	231 (74.0)	186 (61.2)	160 (51.9)	844 (68.8)	
Yes	35 (11.6)	81 (26.0)	118 (38.8)	148 (48.1)	382 (31.2)	
rMED Score (%)						0.220
Low	93 (28.8)	104 (32.2)	86 (26.6)	80 (24.8)	363 (28.1)	
Medium	170 (52.6)	173 (53.6)	183 (56.7)	175 (54.3)	701 (54.3)	
High	60 (18.6)	46 (14.2)	54 (16.7)	67 (20.8)	227 (17.6)	
Kilocalories (x day)	2038.84 (727.46)	2049.65 (702.27)	2026.96 (782.93)	2108.82 (784.83)	2056.03 (749.98)	0.515 ^†^
Alcohol (g/day)	75.06 (130.51)	118.99 (190.88)	160.44 (359.41)	163.39 (203.19)	129.45 (239.08)	<0.001 ^†^
HOMA-IR	1.04 (0.57)	1.41 (0.80)	2.06 (1.92)	2.94 (2.68)	1.89 (1.89)	<0.001 ^†^
Total Cholesterol (mg/dL)	176.63 (30.94)	188.45 (31.81)	194.90 (32.10)	206.09 (39.20)	191.51 (35.28)	<0.001 ^†^
AST (U/L)	20.01 (5.09)	21.06 (6.34)	21.43 (5.41)	24.51 (19.22)	21.75 (10.89)	<0.001 ^†^
ALP (U/L)	47.12 (15.96)	52.10 (13.96)	54.33 (14.57)	58.36 (17.54)	52.97 (16.07)	<0.001 ^†^
Body Mass Index (kg/m^2^)	24.40 (3.76)	26.56 (3.90)	28.79 (4.59)	30.58 (5.51)	27.58 (5.05)	<0.001 ^†^
Triglycerides (mg/dL)	42.64 (12.12)	69.99 (16.17)	100.95 (29.66)	180.37 (85.56)	98.43 (69.30)	<0.001 ^†^
CRP (mg/dL)	0.11 (0.05)	0.15 (0.11)	0.23 (0.25)	0.54 (1.02)	0.26 (0.55)	<0.001 ^†^
FSG (mg/dL)	86.23 (8.43)	91.74 (11.28)	97.55 (14.61)	105.60 (23.92)	95.27 (17.23)	<0.001 ^†^

* As Mean and Standard Deviation (M ± SD) for continuous variables, and frequency and percentage (%) for categorical. Abbreviation: MASLD, Metabolic Dysfunction-associated Steatotic Liver Disease; rMED, Relative Mediterranean Diet; HOMA-IR, Homeostasis Model Assessment of Insulin Resistance; AST, Aspartate Aminotransferase; ALP, Alkaline Phosphatase; CRP, C-reactive protein; FSG, Fasting Serum Glucose. ^^^ Chi-Square or Fisher test when necessary; ^†^ Kruskal–Wallis rank test.

**Table 2:** Logistic regression model of MASLD (yes vs. no) on CTI index both as continuous and categorical.

MASLD	O.R.	s.e. (OR)	*p*-Value	[95% C.I.]	
Model 1 *					
CTI index continue	3.14	0.28	<0.001	2.65	3.74
CTI index quantiles					
Q1 [ref]	1.00	--	--	--	--
Q2	2.32	0.40	<0.001	1.65	3.25
Q3	4.06	0.70	<0.001	2.89	5.70
Q4	9.68	1.78	<0.001	6.75	13.87
Model 2 ^					
CTI index continue	2.46	0.23	<0.001	2.05	2.96
CTI index quantiles					
Q1 [ref]	1.00	--	--	--	--
Q2	1.61	0.30	0.009	1.12	2.31
Q3	2.50	0.46	<0.001	1.74	3.60
Q4	5.62	1.10	<0.001	3.83	8.23
Model 3 †					
CTI index continue	2.43	0.24	<0.001	2.00	2.94
CTI index quantiles					
Q1 [ref]	1.00	--	--	--	--
Q2	1.47	0.28	0.046	1.02	2.13
Q3	2.38	0.46	<0.001	1.62	3.48
Q4	5.16	1.03	<0.001	3.48	7.63
Model 4 ¤					
CTI index continue	2.06	0.23	<0.001	1.65	2.57
CTI index quantiles					
Q1 [ref]	1.00	--	--	--	--
Q2	1.48	0.29	0.049	1.00	2.17
Q3	2.06	0.42	<0.001	1.38	3.08
Q4	3.62	0.82	<0.001	2.32	5.64

Abbreviation: OR, Odds Ratio; se (OR), Standard Error of OR; 95% C.I., Confidence Interval at 95%, MASLD, Metabolic Dysfunction-Associated Steatotic Liver Disease, CTI index, C-reactive protein-Triglycerides-Glucose Index * unadjusted; ^ adjusted for gender and age; † adjusted for gender, age, education, work, marital status, personal annual income and house location; **¤** adjusted for gender, age, education, work, marital status, personal annual income, house location, daily kcal intake, alcohol, smoking habits, HOMA-IR (categorical <2.5 or >2.5), cancer (yes or no), hypertension (yes or no), total cholesterol, AST, ALP.

## Data Availability

The original data presented in this study are openly available in FigShare at https://doi.org/10.6084/m9.figshare.28677308.v3.

## Supplementary Materials

The following supporting information can be downloaded at https://www.mdpi.com/article/10.3390/biomedicines14081860/s1, Table S1: Ultrasound scoring system for the assessment of hepatic steatosis severity; Table S2: Metabolic alterations linked to hepatic steatosis in MASLD affected subjects; Table S3: Variance inflation factor; Table S4: Demographic and clinical characteristics of subject stratified for MASLD; Table S5: Logistic regression models of MASLD (yes vs. no) on CTI index, stratified for age (<65 y.o./>65 y.o.) and gender; Figure S1: Margins plots of logistics regression presented in Table S5.

## Abbreviations

The following abbreviations are used in this manuscript:

SLD	Steatotic Liver Disease
MASLD	Metabolic Dysfunction-Associated Steatotic Liver Disease
NCD	Non-communicable Chronic Disease
NUTRI-HEP	Nutrition and Hepatology
OR	Odds Ratio
C.I.	Confidence Interval
rMED	Relative Mediterranean Diet
CHARLES	China Health and Retirement Longitudinal Study
NHANES	National Health and Nutrition Examination Survey
