# Supplementary material for: The C-Reactive Protein–Triglyceride–Glucose Index as a Biomarker of Metabolic Dysfunction-Associated Steatotic Liver Disease: Results from the Population-Based Italian NUTRI-HEP Cohort
Source: Biomedicines. 2026 Aug 19;14(8):1860. doi: 10.3390/biomedicines14081860 (PMC13509244; doi:10.3390/biomedicines14081860)

Supplementary Materials

Table S1 Ultrasound scoring system for the assessment of hepatic steatosis severity.

Contrast between liver and renal parenchyma	Ultrasound beam penetration	Vascular blurring (especially veins)
Homogeneous echogenicity with no evident liver–kidney contrast (0)	Hepatic parenchyma clearly visible from the surface to the diaphragm (0)	Vascular structures clearly visible (0)
Mild discrepancy in hepatic–renal echogenicity (1)	Attenuation of the ultrasound beam with partial loss of visualization of deep liver structures or diaphragm (1)	Loss of visualization of vascular structures (1)
Wide discrepancy between hepatic and renal (2)	Marked attenuation of the ultrasound beam with loss of visualization of deep liver structures and diaphragm (2)	Vascular structures poorly visible or not visible (2)
Steatosis Score: *Absent (0); Mild (1-2); Moderate (3-5); Severe (6)*		

Table S2 Metabolic alterations linked to hepatic steatosis in MASLD affected subjects.

	**MASLD subjects (n=629)**
**BMI**	
< 25 kg/m^2	87 (13.8%)
≥ 25 kg/m^2	542 (86.2%)
**Fasting Blood Glucose**	
< 100 mg/dL	374 (59.5%)
≥ 100 mg/dL	255 (40.5%)
**Blood Pressure**	
< 130/85 mmHg	506 (80.4%)
≥ 130/85 mmHg	123 (19.6%)
**Serum Triglycerides**	
< 150 mg/dL	466 (74.1%)
≥ 150 mg/dL	163 (25.9%)
**HDL**	
≥ 40 mg/dL (men); 50 mg/dL (women)	371 (59.0%)
< 40 mg/dL (men); 50 mg/dL (women)	258 (41.0%)

Abbreviations: MASLD, Metabolic Dysfunction-Associated Steatotic Liver Disease; BMI, Body Mass Index; HDL, High-Density, Lipoproteins

Table S3. Variance inflation factor.

Variables	VIF
CTI Index Quartiles	
Q1 [ref.]	
Q2	1.658
Q3	1.849
Q4	2.251
Gender	1.282
Age	2.586
Education	1.578
Work	1.348
Marital Status	1.232
Personal annual income	1.059
House location	1.031
Kcal intake (x day)	1.198
Alcohol	1.301
Smoking	1.058
HOMA-IR (<2.5 vs >2.5)	1.178
Cancer	1.045
Hypertension	1.480
Total Cholesterol	1.175
AST	1.057
ALP	1.173
Mean VIF	1.397

Abbreviation VIF, variance inflation factor; AST, Aspartate Aminotransferase; ALP, Alkaline Phosphatase

Table S4. Demographic and clinical characteristics of subject stratified for MASLD (n=1291).

Parameters *	MASLD		Total	p ^
	No (n=668)	Yes (n=629)		
**Age**	49.24±13.80	59.74±12.86	54.33±14.34	<0.001 †
**Quantiles of CTI index (%)**				<0.001
Q1	248 (37.3)	75 (12.0)	323 (25.0)	
Q2	190 (28.6)	133 (21.2)	323 (25.0)	
Q3	145 (21.8)	178 (28.4)	323 (25.0)	
Q4	82 (12.3)	240 (38.3)	322 (24.9)	
**Sex (%)**				<0.001
Female	417 (62.4)	327 (52.0)	744 (57.4)	
Male	251 (37.6)	302 (48.0)	553 (42.6)	
**Education (%)**				<0.001
Primary school	71 (10.6)	211 (33.5)	282 (21.7)	
Secondary school	171 (25.6)	212 (33.7)	383 (29.5)	
High School	307 (46.0)	153 (24.3)	460 (35.5)	
Graduated	119 (17.8)	53 (8.4)	172 (13.3)	
**Work (%)**				<0.001
Managers & Professionals	57 (8.5)	45 (7.2)	102 (7.9)	
Craft, Agricultural and Sales Workers	285 (42.7)	184 (29.3)	469 (36.2)	
Elementary Occupations	93 (13.9)	92 (14.6)	185 (14.3)	
Housewife	74 (11.1)	67 (10.7)	141 (10.9)	
Pensioners	110 (16.5)	215 (34.2)	325 (25.1)	
Jobless	49 (7.3)	26 (4.1)	75 (5.8)	
**Marital Status (%)**				<0.001
Single	115 (17.2)	66 (10.5)	181 (14.0)	
Married or Living together	519 (77.7)	515 (81.9)	1,034 (79.7)	
Separated or Divorced	20 (3.0)	8 (1.3)	28 (2.2)	
Widower	14 (2.1)	40 (6.4)	54 (4.2)	
**Personal annual income (%)**				0.002
<5,000	2 (0.3)	1 (0.2)	3 (0.3)	
5,000-10,000	17 (2.7)	26 (4.5)	43 (3.6)	
10,000-20,000	201 (32.4)	228 (39.4)	429 (35.8)	
20,000-30,000	229 (36.9)	179 (30.9)	408 (34.0)	
30,000-40,000	86 (13.9)	62 (10.7)	148 (12.3)	
40,000-50,000	38 (6.1)	18 (3.1)	56 (4.7)	
>50,000	9 (1.5)	12 (2.1)	21 (1.8)	
No answer	0 (0.0)	1 (0.2)	1 (0.1)	
Do not know	38 (6.1)	52 (9.0)	90 (7.5)	
**House Location (%)**				0.860
Downtown	244 (39.4)	234 (40.3)	478 (39.8)	
Periphery	284 (45.8)	266 (45.9)	550 (45.8)	
Countryside	92 (14.8)	80 (13.8)	172 (14.3)	
**Smoke**				0.872
No	587 (87.9)	550 (87.6)	1,137 (87.7)	
Yes	81 (12.1)	78 (12.4)	159 (12.3)	
**Cancer (%)**				0.153
No	608 (96.2)	566 (94.5)	1,174 (95.4)	
Yes	24 (3.8)	33 (5.5)	57 (4.6)	

**Hypertension (%)**				<0.001
No	517 (81.8)	330 (55.0)	847 (68.8)	
Yes	115 (18.2)	270 (45.0)	385 (31.2)	
**rMED Score (%)**				0.460
Low	196 (29.3)	169 (26.9)	365 (28.1)	
Medium	362 (54.2)	343 (54.5)	705 (54.4)	
High	110 (16.5)	117 (18.6)	227 (17.5)	
**Kilocalories (x day)**	2,100.33±724.88	2,009.46±774.05	2,056.26±750.22	0.002 †
**Alcohol (g/day)**	113.73 (265.46)	146.79 (205.59)	129.76 (238.79)	0.006 †
**HOMA-IR**	1.33 (0.90)	2.43 (2.38)	1.89 (1.88)	<0.001 †
**Alkaline Phosphatase (U/L)**	50.11 (15.56)	56.04 (16.11)	52.98 (16.10)	<0.001 †
**AST (U/L)**	20.70 (5.94)	22.85 (14.29)	21.74 (10.87)	<0.001 †
**Total Cholesterol (mg/dL)**	188.90 (33.06)	193.96 (37.50)	191.35 (35.36)	0.011 †
**BMI (kg/m^2)**	25.040±3.588	30.285±4.968	27.584±5.046	<0.001 †
**Triglycerides (mg/dL)**	80.727±58.552	117.222±74.602	98.410±69.233	<0.001 †
**C-Reactive Protein (mg/dL)**	0.21 (0.52)	0.31 (0.58)	0.26 (0.55)	<0.001 †
**Fasting Blood Glucose (mg/dL)**	90.13 (10.54)	100.89 (21.06)	95.34 (17.34)	<0.001 †

* *As Mean and Standard Deviation (M±SD) for continuous variable, and frequency and percentage (%) for categorical.*

Abbreviation: MASLD, Metabolic Dysfunction-associated Steatotic Liver Disease; BMI, body mass index; rMED, Relative Mediterranean Diet, AST, Aspartate Aminotransferase. ^ Chi-Square or Fisher test when necessary; † Wilcoxon rank-sum test.

Table S5. Logistic regression models of MASLD (yes vs. no) on CTI index, stratified for age (<65 y.o./>65 y.o.) and gender.

MASLD	O.R.	s.e. (OR)	p-value	[95% C. I.]	
**CTI over age65 ***					
Q1 [ref.]	1.00	--	--	--	--
Q2	2.16	0.43	<0.001	1.46	3.19
Q3	3.73	0.75	<0.001	2.51	5.54
Q4	9.85	2.15	<0.001	6.42	15.10
**CTI over gender ^**					
Q1 [ref.]	1.00	--	--	--	--
Q2	1.86	0.41	0.005	1.20	2.88
Q3	3.53	0.80	<0.001	2.27	5.50
Q4	7.86	1.96	<0.001	4.83	12.80

Abbreviation: OR, Odds Ratio; se (OR), Standard Error of OR; 95% C.I., Confidence Interval at 95%.

* adjusted for age (<65 y.o./>65 y.o.) and gender, stratified over age

^ adjusted for age (<65 y.o./>65 y.o.) and gender, stratified over gender

Figure S1. Margins plots of logistics regression presented in table S5.

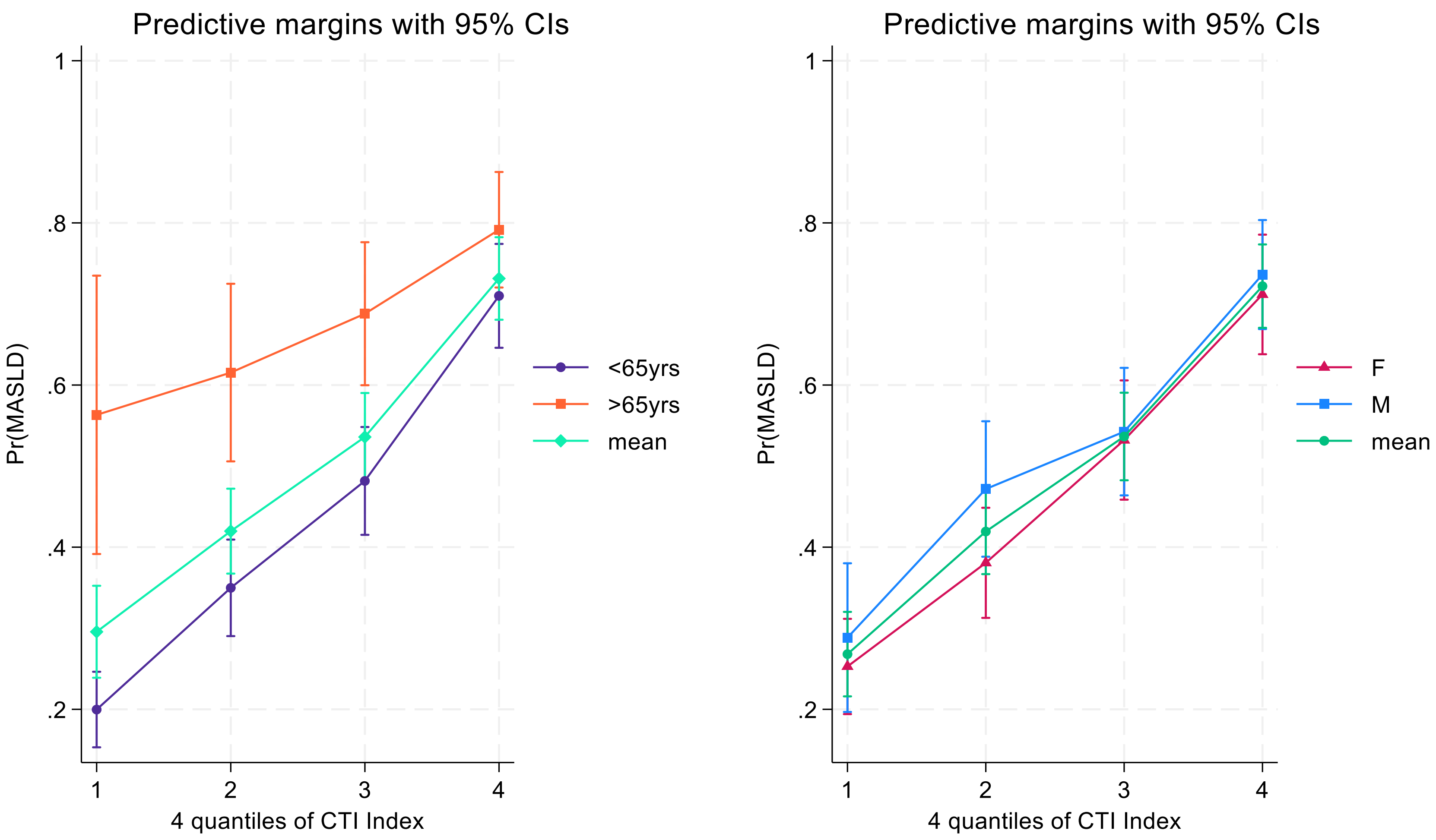

Supplement: Supplementary file 1 [file biomedicines-14-01860-s001.zip › biomedicines-4497036-supplementary.pdf]
